# Effects of anthocyanin-rich Napier grass silage on feed intake, milk production, plasma profile, and nutritional digestibility in lactating crossbred Saanen goats

**DOI:** 10.14202/vetworld.2024.2802-2810

**Published:** 2024-12-14

**Authors:** Anan Chaokaur, Janjira Sittiya, Pornpan Saenphoom, Pattaraporn Poommarin, Wilasinee Inyawilert, Jai-Wei Lee, Attapol Tiantong

**Affiliations:** 1 Program in Animal Science, Faculty of Animal Sciences and Agricultural Technology, Silpakorn University, Phetchaburi IT Campus, Cha-Am, Phetchaburi, 76120, Thailand; 2 Department of Agricultural Science, Faculty of Agriculture Natural Resources and Environment, Naresuan University, Phitsanulok, 65000, Thailand; 3 Department of Tropical Agriculture and International Cooperation, National Pingtung University of Science and Technology, Pingtung, 91201, Taiwan

**Keywords:** anthocyanin, antioxidant, lactating goat, milk yield, nutrient digestibility

## Abstract

**Background and Aim:**

Anthocyanins are potent antioxidants and scavengers of free radicals found in fruits, purple corn, and other naturally occurring purple plants. Several studies have demonstrated that anthocyanins possess strong antioxidant properties and can enhance ruminant production by modulating intracellular oxidative stress and modifying ruminal fermentation. This study aimed to examine the effects of anthocyanin-rich Napier grass silage (ANS) on feed intake, nutrient digestibility, milk production, plasma profile, and antioxidant capacity in lactating crossbred Saanen goats.

**Materials and Methods:**

Nine healthy lactating goats (crossbreeds of Thai-native and Saanen breeds aged 8–12 months) were selected and randomly assigned to one of three experimental diets: (1) Corn silage (CS), (2) Napier grass silage (NS), and (3) ANS. Daily feed refusals were recorded to calculate dry matter intake for the animal performance assessment. In addition to analyzing feed intake, nutrient intake, and nutrient digestibility, milk and blood samples were also analyzed for protein composition, leukocyte count, and antioxidant capacity.

**Results:**

CS-fed goats had the highest (p < 0.05) feed intake (1.09 kg/day), a value markedly exceeding those of NS- and ANS-fed goats (0.80 and 0.76 kg/day, respectively). They also had higher (p < 0.05) organic matter (OM) and neutral detergent fiber intake (0.99 kg and 0.50 kg/day) than the other two groups. Protein intake did not differ significantly (0.14, 0.12, and 0.12 kg/day for CS, NS, and ANS, respectively). The CS group showed higher (p < 0.05) dry matter and OM digestibility (69.42% and 69.83%) than the NS and ANS groups, which had lower (p < 0.05) fiber digestibility. Regarding milk production, the CS and ANS groups produced 1.15 and 1.16 kg/day, respectively, whereas the NS group produced 1.11 kg/day. No significant differences in leukocyte counts were observed. Furthermore, the CS group exhibited the highest (p < 0.05) superoxide dismutase inhibition (16.05%) on day 35, whereas the ANS group showed the highest (p < 0.05) total antioxidant capacity on multiple days (21, 35, 49, and 63).

**Conclusion:**

ANS can be a valuable component of dairy goat diets, particularly in regions prone to heat-induced oxidative stress.

## Introduction

In 2023, Thailand’s goat population was estimated at 1.5 million. Most of these goats are raised by smallholders, with around 90,204 households engaged in goat farming as a supplementary occupation. Several factors, including genotype or breed, nutrition, age, weight, sex, management, and the environment, influence goat growth and body composition. Dairy goat milk production depends on breed, dam parity, number of kid, nutrition, management, and environmental conditions [1].

Heat-induced oxidative stress has recently emerged as a significant performance-limiting factor in animals [2]. Heat stress intensifies oxidative stress by increasing free radical production while reducing the body’s ability to neutralize it [3]. This leads to a cascade of detrimental impacts on animal performance and health, such as reduced feed intake [4], weight loss [5], diminished milk production, heightened disease susceptibility, and reproductive issues [6]. In tropical regions where heat stress is common, oxidative stress is a notable concern, affecting animal performance and health.

Growing interest in natural phenolic compounds, including anthocyanins, stems for their potential health benefits. These compounds have been explored as potential substitutes for antibiotics and synthetic growth enhancers in sustainably produced animal feed. Anthocyanins, known for their strong antioxidant potential [7, 8], can neutralize free radicals, thereby reducing oxidative damage to cells, tissues, proteins, membranes, and mitochondria [9]. Research on anthocyanin supplementation in ruminants has shown promising outcomes, suggesting benefits for animal health, milk quality, and productivity. Incorporating anthocyanin-rich plants or their byproducts into dairy goat diets is a particularly relevant strategy in tropical climates where heat stress constrains animal performance and health [10, 11].

Purple Napier grass (*Pennisetum purpureum*), a semi-dwarf cultivar developed by the USDA and the University of Georgia, was imported to Thailand by the Dairy Farming Promotion Organization of Thailand [12]. This grass species contains anthocyanins known for their antioxidant properties and potential health benefits. Studies by Suong *et al*. [8], Purba *et al*. [13], and Suong *et al*. [14] have indicated that anthocyanin-rich diets can increase the levels of important antioxidant enzymes, such as superoxide dismutase (SOD), glutathione peroxidase, and catalase, in lactating dairy goats.

Onjai-Uea *et al*. [15] found that purple Napier grass exhibited markedly higher crude protein (CP) content and anthocyanin concentration than Napier Pakchong 1. However, published studies on the impact of anthocyanin-rich Napier grass silage (ANS) on dairy goat output are lacking. This study aimed to examine the effects of ANS on feed intake, nutrient digestibility, milk production, plasma profile, and antioxidant capacity of lactating crossbred Saanen goats.

## Materials and Methods

### Ethical approval

The protocols for animal experimentation were approved by the Institutional Animal Care and Use Committee at Silpakorn University, Thailand. All animal experiments adhered to the criteria outlined in the Ethics of Use to Animals for Scientific Work by the National Research Council of Thailand (Project ID: 03/2562).

### Study period and location

This study was conducted from June 2019 to December 2020 at the Faculty of Animal Sciences and Agricultural Technology Farm at the Silpakorn University Phetchaburi IT campus in Thailand.

### Animals

Nine healthy lactating goats, crossbreeds of Thai-native and Saanen breeds, were selected (average weight: 39.38 kg [±4.56 standard deviation]; average age: 8–12 months). Using a completely randomized design, the goats were individually housed and randomly assigned to one of three experimental diets (Table-1): (1) Corn silage (CS), (2) Napier grass silage (NS), and (3) ANS. All goats received *ad libitum* complete roughage along with concentrated feed containing a protein level of not <18% CP, equivalent to 1.5% of their body weight per day. This nutritional provision was calculated to meet the goats’ nutritional needs according to NRC standards [16]. The goats underwent a 14-day acclimation period to adjust to nutrition management before data collection. The animals were gradually introduced to the experimental diets throughout the acclimation phase. After 14 days, they received the experimental diets daily for 63 days. During the experiment, feeding was offered at 07:00 and 16:00 daily. The goats had unrestricted access to potable water and a trace mineral salt block. Feed refusals were recorded daily and used to calculate dry matter intake (DMI) to assess animal performance.

**Table-1:** Chemical compositions of the concentrates and roughages used in the experiments.

Components (%)	Concentrate	Roughages^1)^		

		CS	NS	ANS
DM	90.43	23.04	18.70	22.66

	**DM basis (%)**			

OM	90.65	92.26	86.76	85.97
CP	18.15	6.43	6.82	6.31
Ether extract	4.12	2.71	1.54	1.46
Crude fiber	9.86	28.2	26.63	28.33
Ash	9.35	7.74	13.24	14.03
NDF	32.62	61.62	65.02	67.38
ADF	21.35	34.48	37.42	38.66
Acid detergent lignin	3.21	3.81	3.83	4.06
Hemicellulose	11.27	27.14	27.60	28.72
Cellulose	18.14	30.67	33.59	34.60
Anthocyanin (mg/100 g DM)	-	0.11	0.17	8.61

1) CS=Corn silage, NS=Napier grass silage, ANS=Anthocyanin-rich Napier grass silage, DM=Dry matter, NDF=Neutral detergent fiber, ADF=Acid detergent fiber, OM=Organic matter, CP=Crude protein

### Preparation of silage and sampling

We prepared Napier grass and anthocyanin-rich Napier grass at the Phetchaburi Animal Feed Research and Development Center, Cha-am District, Phetchaburi, Thailand. This was achieved by planting one plot of Napier grass and one plot of anthocyanin-rich Napier grass. The first cutting was performed after 60 days and silage was prepared. After fermenting for a month, the silage tank was opened to randomly collect samples of fermented grass, which were then analyzed for moisture, dry matter (DM), and total anthocyanin content.

CS preparation followed a similar procedure to that used to prepare Napier and anthocyanin-rich Napier grass. However, the corn was cut on 70^th^ day and then processed to produce silage by harvesting fresh (green) material, compacting it, and storing it in a bag for fermentation under regulated conditions. CS, NS, and ANS samples were randomly collected after 1 month of fermentation from various locations within each silage tank, amounting to approximately 5 kg of silage samples. Each sample was divided into two parts for moisture analysis and total anthocyanin content determination.

### Extraction and analysis of anthocyanins

The total anthocyanin content of the roughage samples (CS, NS, and ANS) was analyzed using acid as the solvent. The total anthocyanin content was analyzed using the differential pH method, measuring absorbance at specific wavelengths (538 and 700 nm), following standard methods as described by Fuleki and Francis [17, 18]. The CS, NS, and ANS samples were cut into small pieces and ground into a fine paste using a blender. Twenty grams of each sample (three replicates) were placed in a beaker. Then, 100 mL of the extraction solvent (comprising 80 mL of methanol, 0.1 mL of hydrochloric acid, and 19.9 mL of distilled water) were added. The procedure included sample soaking, gentle shaking for thorough mixing, and wrapped in aluminum foil for light protection. The mixture was refrigerated at 4°C for 12 h. Subsequently, the samples were filtered through filter paper (No. 1, 125 mm size) using a vacuum filter. The filtered solution was then adjusted to a pH 1 using 0.1% hydrochloric acid. The resulting solution was used to measure absorbance at wavelengths between 538 and 700 nm [19, 20].

### Measurement of feed and nutrient intakes and digestibility

Samples of concentrate and roughage were randomly collected every 2 weeks during the experiment until the last week. Both samples were baked at 60°C for 72 h and ground using a 1-mm sieve. This process was conducted to analyze the chemical composition of the experimental feed used in the study, including DM, ash, CP, and acid-insoluble ash, according to the Association of Official Analytical Chemists method [21]. In addition, the fiber components, including neutral detergent fiber (NDF), acid detergent fiber (ADF), and acid detergent lignin, were analyzed using a method described by Goering and Van Soest [22] to assess nutrient intake.

Fecal samples from all goats were collected on three consecutive days during the last week of the experiment using the gap sampling method (collected from the anus). The samples were then baked at 60°C for 72 h, ground through a 1-mm sieve, and analyzed for the chemical composition of the feces. Standard methods similar to those used in feed analysis were used to evaluate nutrient digestibility.

### Blood and milk sampling

The blood sample was obtained through jugular venipuncture into a 10 mL heparin-containing vacuum tube before morning feeding at 07:00 on days 0, 7, 21, 35, 49, and 63. The heparinized blood samples were centrifuged at 4°C for 30 min at 1,000× *g*. In addition, aliquots of the recovered plasma were stored at −20°C to assess both antioxidant activity and protein concentration. The leukocyte count, which was determined using a microscope, was used to assess animal health. In parallel, 25 mL milk sample was manually and aseptically collected from each quarter on days 0, 7, 21, 35, 49, and 63. The milk samples were preserved in an ice bath until they were fractionated for 20 min at 4°C by centrifugation at 400× *g*. The somatic cell count (SCC) of raw milk was determined using a Lactoscan Somatic Cells Counter (Milkotronic Ltd., Nova Zagora, Bulgaria) before milk fractionation. Subsequently, small aliquots of the skimmed cell-free supernatant were obtained to determine the total milk protein concentration.

### Measurement of plasma and milk protein concentrations

Total protein concentrations in both plasma and milk were measured using a Bio-Rad protein assay following the Bradford method (Bio-Rad Laboratories, Hercules, CA, USA). The assay was carried out in microplate format (SPECTRO-Star Nano, BMG Labtech GmbH, Ortenberg, Germany), and a standard curve was constructed using BSA (Sigma-Aldrich) to calculate the required volume for sodium dodecyl sulfate-polyacrylamide gel electrophoresis (SDS-PAGE).

### Measurement of plasma protein composition

Using the Laemmli sample buffer coupled with a 7% separating gel, 2× Native Sample Buffer (161–0738, Bio-Rad Laboratories) was mixed with the equivalent of 10 g of protein obtained from the plasma before performing SDS-PAGE. Staining was performed using Coomassie blue dyes (161–0786, Bio-Rad Laboratories) for 60 min. Subsequently, to improve band visibility, the samples were preserved in distilled water. Quantification was performed using Image Lab V.6.1.0 Build 7 (Bio-Rad Laboratories), from which relevant band images were acquired using a GelDoc Go Gel Imaging System (Bio-Rad Laboratories). The expected band sizes for the proteins were as follows: 110–150 kDa for globulin and 50–60 kDa for albumin.

### Measurement of antioxidant activity

The plasma levels of SOD and total antioxidant capacity (TAC) were enzymatically determined in duplicate using the SOD Activity Assay Kit (ab65329, Abcam, Cambridge, UK) and the TAC Assay Kit (ab65354, Abcam), respectively. The assays were conducted in microplates (96 wells, UV plate), and absorption monitoring was performed using a microplate reader (SPECTRO-Star Nano, BMG Labtech GmbH, Ortenberg, Germany) as per manufacturer’s instructions.

### Statistical analysis

The statistical analysis was conducted using a two-way analysis of variance (ANOVA) to assess differences among treatment groups. Following ANOVA, Duncan’s multiple range tests were used to compare means, with a significance level set at p < 0.05. All analyses were performed using the R statistical package [23], and results are presented as the mean ± standard deviation.

## Results

### Chemical composition

An examination of the chemical composition of the concentrate and roughage (Table-1) revealed that the concentrated feed used for the experimental goats contained DM, protein, fat, fiber, NDF, and ADF, with values of 90.43%, 18.15%, 4.12%, 9.86%, 32.62%, and 21.35%, respectively. The CS group exhibited DM, protein, fat, fiber, NDF, and ADF contents of 23.04%, 6.43%, 2.71%, 28.2%, 61.62%, and 34.48%, respectively – these values were 18.70%, 6.82%, 1.54%, 26.63%, 65.02%, and 37.42% for the NS group and 22.66%, 6.31%, 1.46%, 28.33%, 67.38%, and 38.66% for the ANS group. Table-1 presents the analysis and comparison results of the total anthocyanin content in the roughage samples used to feed the goats. The ANS group had the highest total anthocyanin content compared with the NS and CS groups. The values were 8.61, 0.17, and 0.11 mg/100 g DM, respectively.

### Feed intake

The analysis of feed intake by goats in each experimental group (Table-2) revealed no significant variation in concentrated feed intake among the goats (p > 0.05). On average, the animals consumed 0.59 kg of feed per day, which accounted for 1.51% of their body weight, or a mean of 37.70 g/kg of metabolic weight (kgW^0.75^). For roughage intake, the experimental diet resulted in significant variation (p < 0.01) in the intake of each group of goats. In particular, goats consumed the greatest quantity of CS as roughage more than NS or ANS (p < 0.01): the mean values, calculated as the quantity of roughage consumed daily, were 0.48, 0.20, and 0.19 kg per animal per day. The CS, NS, and ANS groups had averages of 1.21%, 0.49%, and 0.49% of body weight, or 30.35, 12.46, and 12.25 g/kg of metabolic weight (kgW^0.75^), respectively. Therefore, the total amount of feed intake in the CS group was the highest among the experimental groups, with a significant difference (p < 0.05) and a mean value of 1.09 kg/day, representing an average of 2.72% of body weight and calculated on average as 68.30 g/kg of metabolic weight (kgW^0.75^).

**Table-2:** Feed intake of CS, NS, and ANS-fed goats.

Feed intake	Dietary treatments			SEM	p-value

	CS	NS	ANS		
Roughage intake					
kg/day	0.48 ± 0.06^a^	0.20 ± 0.03^b^	0.19 ± 0.02^b^	0.014	<0.01
% BW	1.21 ± 0.02^a^	0.49 ± 0.01^b^	0.49 ± 0.01^b^	0.005	<0.01
g/kgW^0.75^	30.35 ± 1.46^a^	12.46 ± 0.58^b^	12.25 ± 0.21^b^	0.306	<0.01
Concentrate intake					
kg/day	0.61 ± 0.06	0.60 ± 0.10	0.57 ± 0.06	0.026	0.865
% BW	1.51 ± 0.01	1.51 ± 0.01	1.50 ± 0.01	0.004	0.770
g/kgW^0.75^	37.96 ± 0.79	37.81 ± 1.84	37.34 ± 1.11	0.441	0.843
Total feed intake					
kg/day	1.09 ± 0.12^a^	0.80 ± 0.14^b^	0.76 ± 0.08^b^	0.039	0.025
% BW	2.72 ± 0.02^a^	2.00 ± 0.02^b^	2.00 ± 0.01^b^	0.005	<0.01
g/kgW^0.75^	68.30 ± 2.26^a^	50.27 ± 2.42^b^	49.60 ± 1.31^b^	0.685	<0.01

^a,b^Means within rows followed with different superscript letters are statistically different (p < 0.05). BW=Body weight, W^0.75^=Metabolic weight, CS=Corn silage, NS=Napier grass silage, ANS=Anthocyanin-rich Napier grass silage, SEM=Standard error of the mean

### Nutrient intake

The nutrient intake analysis of goats in each experimental group (Table-3) revealed that goats fed CS as a source of roughage had a higher total feed intake than those in the other experimental groups. Consequently, the CS group had significantly higher organic matter (OM) and NDF fiber intake than the other groups (p < 0.01), with values of 0.99 and 0.50 kg of nutrients per head per day, respectively. Similarly, the CS group had markedly higher ADF intake than the other groups (p < 0.05).

**Table-3:** Nutrient intake of CS, NS, and ANS-fed goats.

Nutrients (kg/day)	Dietary treatments			SEM	p-value

	CS	NS	ANS		
DMI	1.09 ± 0.12^a^	0.80 ± 0.14^b^	0.76 ± 0.08^b^	0.039	0.025
OMI	0.99 ± 0.11^a^	0.72 ± 0.13^b^	0.68 ± 0.07^b^	0.107	0.023
CPI	0.14 ± 0.02	0.12 ± 0.02	0.12 ± 0.01	0.018	0.444
NDFI	0.50 ± 0.06^a^	0.33 ± 0.06^b^	0.31 ± 0.03^b^	0.052	0.008
ADFI	0.30 ± 0.03^a^	0.20 ± 0.04^b^	0.20 ± 0.02^b^	0.031	0.012

^a,b^Means within rows followed with different superscript letters are statistically different (p < 0.05). DMI=Dry matter intake, OMI=Organic matter intake, CPI=Crude protein intake, NDFI=Neutral detergent fiber intake, ADFI=Acid detergent fiber intake, CS=Corn silage, NS=Napier grass silage, ANS=Anthocyanin-rich Napier grass silage, SEM=Standard error of the mean

However, the total CP intake from feed among the experimental goats did not show a significant difference (p > 0.05), with values of 0.14, 0.12, and 0.12 kg of protein per head per day for goats fed CS, NS, and ANS, respectively.

### Nutrient digestibility

The nutrient digestibility analysis of feed in each experimental group (Table-4) revealed higher dry and OM digestibility in the CS group than in the NS and ANS groups (p < 0.01), with average values of 69.42%, 62.29%, and 61.80% DM digestibility in the CS, NS, and ANS groups, respectively. The average OM digestibility values were 69.83%, 62.83%, and 63.35%, respectively. Fiber digestibility in goats’ feed differed significantly among the experimental groups. The digestibility of NDF and ADF digestibility exhibited significant differences (p < 0.01 and p < 0.05, respectively). The NS and ANS groups had lower NDF and ADF digestibility than the CS group.

**Table-4:** Nutrient digestibility of CS, NS, and ANS-fed goats.

Nutrient digestibility (%)	Dietary treatments			SEM	p-value

	CS	NS	ANS		
DM	69.42 ± 0.60^a^	62.29 ± 0.52^b^	61.80 ± 0.60^b^	0.193	<0.01
OM	69.83 ± 0.68^a^	62.83 ± 0.54^b^	63.35 ± 0.50^b^	0.192	<0.01
CP	60.67 ± 0.41^a^	56.44 ± 0.55^b^	56.37 ± 0.59^b^	0.174	<0.01
NDF	64.33 ± 0.56^a^	54.51 ± 0.70^b^	53.82 ± 0.63^b^	0.212	<0.01
ADF	45.19 ± 0.50^a^	43.40 ± 0.54^b^	43.54 ± 0.63^b^	0.188	0.14

^a,b^Means within rows followed with different superscript letters are statistically different (p < 0.05). DM=Dry matter, OM=Organic matter, CP=Crude protein, NDF=Neutral detergent fiber, ADF=Acid detergent fiber, CS=Corn silage, NS=Napier grass silage, ANS=Anthocyanin-rich Napier grass silage, SEM=Standard error of the mean

In addition, the protein digestibility values differed significantly (p < 0.01) among the CS, NS, and ANS groups, with average values of 60.67%, 56.44%, and 56.37%, respectively.

### Milk yield

**Figure-1:**
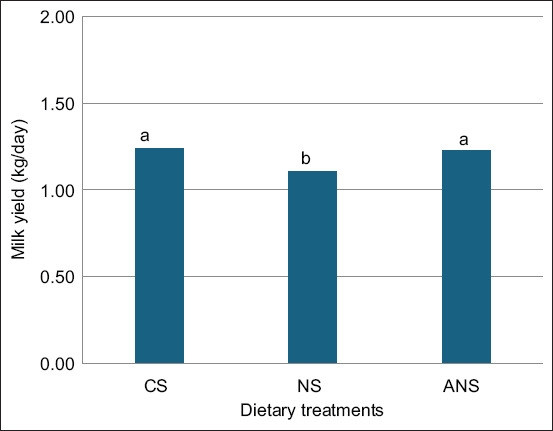
Milk yield of goats fed CS, NS, and ANS. CS=Corn silage, NS=Napier grass silage, ANS=Anthocyanin-rich Napier grass silage. ^a,b^Values with different superscript letters differ significantly (p < 0.05).

The milk yield analysis revealed that the CS and ANS groups had average daily milk yields of 1.15 and 1.16 kg/day, respectively, which were significantly higher (p < 0.05) than the NS group’s yield of 1.11 kg/day (Figure-1).

### Blood biochemical parameters and milk composition

No differences (p > 0.05) were observed in the leukocytes of goats fed different roughage diets among the three experimental groups. The SCCs in the milk analyzed on day 63 of the experiment followed the order of 137.73 × 10^4^, 88.28 × 10^4^, and 106.57 × 10^4^ cells/mL for the NS, CS, and ANS groups, respectively (Table-5).

**Table-5:** Blood biochemical parameters and milk composition of CS, NS, and ANS-fed goats.

Parameters	Dietary treatments			SEM	p-value

	CS	NS	ANS		
Blood parameter					
Leukocytes (×10^4^ cells/mL)					
Day 0	376.17 ± 69.73	398.20 ± 138.91	405.50 ± 80.97	29.54	0.935
Day 7	379.17 ± 169.71	368.67 ± 12.10	454.77 ± 28.56	31.79	0.548
Day 21	402.83 ± 83.52	342.10 ± 44.01	449.87 ± 22.25	22.50	0.140
Day 35	436.17 ± 91.18	387.67 ± 34.59	475.33 ± 62.20	23.07	0.340
Day 49	432.83 ± 71.80	397.00 ± 9.17	489.50 ± 57.41	20.45	0.182
Day 63	383.73 ± 23.84	416.67 ± 61.17	347.17 ± 47.90	16.86	0.262
Plasma protein (g/L)					
Day 0	83.26 ± 16.72	89.63 ± 3.34	95.67 ± 5.02	3.46	0.295
Day 7	95.17 ± 5.40	90.19 ± 5.24	87.87 ± 5.14	1.86	0.837
Day 21	67.91 ± 12.18	68.62 ± 2.01	64.51 ± 9.25	2.65	0.837
Day 35	58.14 ± 3.29	61.61 ± 13.29	56.24 ± 13.90	3.35	0.844
Day 49	64.35 ± 2.02	63.56 ± 3.00	70.14 ± 12.85	2.46	0.554
Day 63	66.21 ± 2.78	68.30 ± 3.13	66.46 ± 3.17	0.93	0.670
Milk parameter					
SCC (×10^4^ cells/mL)					
Day 0	35.07 ± 10.92	46.49 ± 11.32	39.63 ± 22.32	4.84	0.687
Day 7	42.60 ± 16.24	51.67 ± 31.72	49.63 ± 7.41	6.22	0.861
Day 21	47.13 ± 7.22	56.95 ± 18.72	63.73 ± 13.24	4.67	0.396
Day 35	77.13 ± 9.03	74.56 ± 20.92	64.44 ± 9.84	4.57	0.552
Day 49	63.52 ± 22.44	94.90 ± 15.25	81.32 ± 4.66	6.46	0.129
Day 63	88.28 ± 9.20^b^	137.73 ± 12.43^a^	106.57 ± 5.55^b^	7.72	<0.01
Total protein (g/L)					
Day 0	31.17 ± 2.24	29.60 ± 1.75	30.68 ± 3.93	0.84	0.788
Day 7	40.11 ± 0.30^a^	32.56 ± 2.18^b^	38.60 ± 0.24^a^	1.21	<0.01
Day 21	39.87 ± 0.30^a^	31.59 ± 3.99^b^	39.57 ± 0.85^a^	1.52	<0.01
Day 35	42.22 ± 0.85^a^	34.25 ± 5.20^b^	44.34 ± 2.60^a^	1.82	0.024
Day 49	42.28 ± 1.15^a^	28.93 ± 2.78^b^	39.26 ± 2.72^a^	2.13	<0.01
Day 63	41.32 ± 3.08^a^	35.28 ± 4.89^ab^	34.07 ± 1.63^b^	1.50	0.087

^a,b^Means within rows followed by different superscript letters differ significantly (p < 0.05), CS=Corn silage, NS=Napier grass silage, ANS=Anthocyanin-rich Napier grass silage, SEM=Standard error of the mean.

A comparison of the total protein concentrations in the plasma and milk among the CS, NS, and ANS groups revealed no significant differences in plasma protein levels among the three experimental groups.

### Plasma protein content

**Figure-2:**
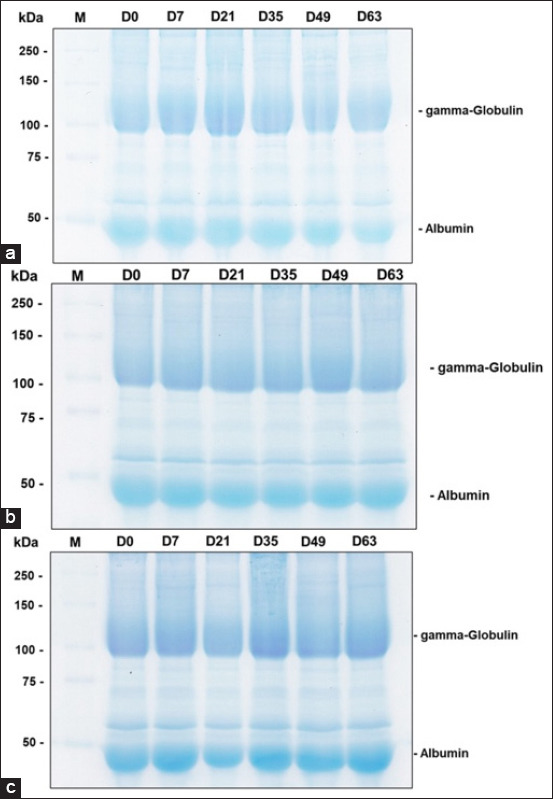
Images of gamma-globulin and albumin bands in the raw milk of goats fed (a) corn silage, (b) Napier grass silage, and (c) anthocyanin-rich Napier grass silage.

**Figure-3:**
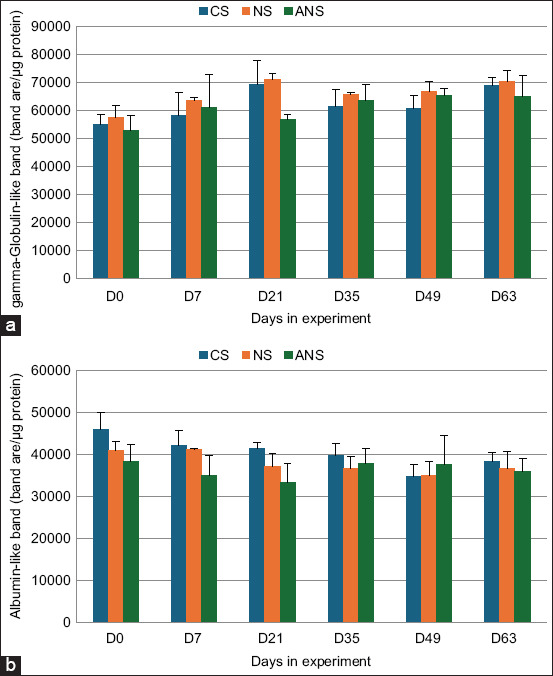
Abundance of (a) gamma-globulin and (b) albumin in raw milk from goats fed CS, NS, and ANS. CS=Corn silage, NS=Napier grass silage, ANS=Anthocyanin-rich Napier grass silage.

Analysis of the identified protein bands in plasma using the SDS-PAGE method and the Image Lab program to determine the band area revealed no significant differences among the CS, NS, and ANS groups. Most plasma proteins measuring 150 kDa in weight were classified as gamma-globulins, whereas proteins with a molecular weight of 50 kDa were classified as albumin proteins. Most of both protein types are present in blood components (Figures-2 and 3).

### Antioxidant capacity in plasma

**Figure-4:**
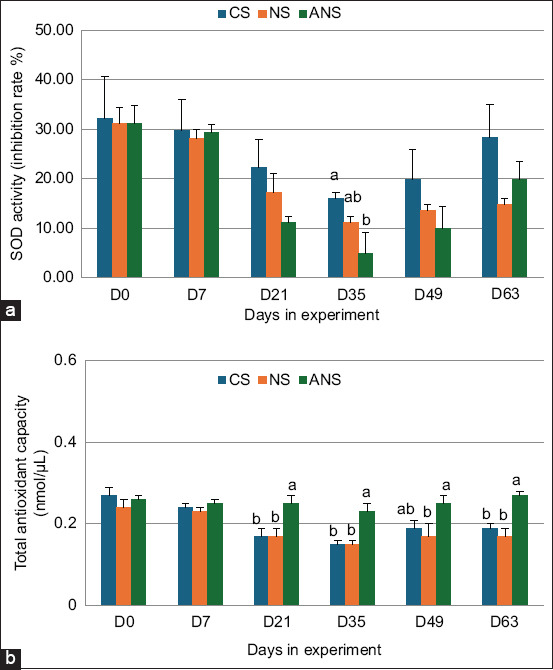
(a) Superoxide dismutase activity and (b) Antioxidant activity in the plasma of CS, NS, and ANS-fed goats. CS=Corn silage, NS=Napier grass silage, ANS=Anthocyanin-rich Napier grass silage. ^a,b^Values with different superscript letters differ significantly (p < 0.05).

Figure-4 shows the SOD and TAC results. On day 35, the CS group showed the highest significant SOD inhibition percentage of 16.05%, whereas the NS and ANS groups showed SOD inhibition percentages of 11.11% and 4.94%, respectively. The ANS group had a significantly higher TAC than the CS and NS groups on days 21, 35, 49, and 63.

## Discussion

The present study evaluated the effects of ANS on feed intake, milk production, and nutrient digestibility in crossbred Saanen goats. The findings provide insights into the potential benefits of incorporating ANS into dairy goat diets, particularly in tropical regions where heat stress and oxidative stress are prevalent.

The analysis of the chemical composition of roughage using purple NS revealed that roughage in the ANS group, which was used to feed the experimental goats, increased crude fiber, NDF, ADF, and anthocyanin levels compared with the other treatments. However, the OM, NDF, and ADF levels were similar to those reported by Onjai-uea *et al*. [15], who also used purple NS to replace Napier Pakchong 1 grass silage at 50% and 100%. The anthocyanin content found in this experiment was only 8.61 mg/100 g DM, whereas Onjai-uea *et al*. [15] reported anthocyanin contents of 118.06 mg/g DM in the purple NS group and 81.37 mg/g DM in the group with 50% replacement. Our study found that the DM and CP levels in the ANS group were 22.66% and 6.31%, respectively. In contrast, Onjai-uea *et al*. [15] reported 35.67% DM and 17.49% CP in the purple NS group.

The goats fed CS had the highest total feed intake compared to those fed NS or ANS. The significant variation in roughage intake among the groups suggests that CS is more palatable or better accepted by goats, leading to higher intake levels. However, the lack of a significant difference in concentrated feed intake among the groups indicates that the primary variation in total feed intake can be attributed to the roughage component. These results align with previous findings that goats prefer roughage over other types, possibly due to differences in texture, taste, and nutrient content.

Jöbstl *et al*. [24] reported that anthocyanins, which are phenolic compounds, impart a bitter taste to plants, potentially reducing palatability and lowering feed intake. This aligns with the study by Onjai-uea *et al*. [15], which tested dairy goats fed roughage mixtures of ensiled Napier Pakchong 1 grass and purple NS at ratios of 100:0, 50:50, and 0:100. They found that increasing purple NS levels resulted in decreased total feed intake, with values of 1.21, 1.16, and 1.13 kg/head/day, respectively.

The markedly higher OM and NDF fiber intake observed in the CS group compared with the NS and ANS groups may have resulted from the higher CS palatability and digestibility, facilitating greater nutrient absorption. Interestingly, while the total CP intake did not differ significantly among the groups, nutrient digestibility was notably higher in the CS group. This suggests that roughage can impact nutrient utilization efficiency, with CS promoting better overall digestibility. Tian *et al*. [11] reported that purple CS did not affect the digestibility of DM, CP, or crude fiber in goats.

Milk yield was marginally higher in the ANS group than in the NS group, with the CS group producing slightly less milk than the ANS group. Although the differences in milk yield were not statistically significant, the trend suggests that ANS supports milk production to a similar extent as CS. This finding is particularly relevant for sustainable animal husbandry, as ANS could be a viable alternative to traditional silage, offering potential antioxidant benefits without compromising milk yield.

The results are similar to the study by Onjai-uea *et al*. [15], which found that goats fed a diet containing 100% purple NS showed higher milk production levels, particularly in terms of lactose, compared to goats fed other types of roughage. In addition, Harvatine and Allen [25] reported that increasing DMI corresponds to an increase in milk yield and milk protein.

The absence of significant differences in leukocyte counts or total protein levels in plasma and milk among the three groups indicates that roughage did not adversely affect health or metabolic status of goats. However, the NS group exhibited the highest SCC in milk, suggesting the potential for subclinical mastitis or other udder health issues. In contrast, the CS and ANS groups had lower SCC values, indicating better udder health and milk quality. The correlation between alterations in SCC and the onset of mastitis is well established, with numerous studies identifying an increase in SCC as an indicator of mastitis. Nagahata *et al*. [26] reported that SCC levels in goat milk are related to the health status of the mammary gland.

Purple NS was associated with reduced SCC in milk, suggesting a potentially beneficial relationship between antibacterial activity and increased anthocyanin consumption. Anthocyanins can inhibit bacterial cell wall activity, leading to cytoplasm leakage and thereby significantly restricting bacterial growth [15, 27]. The antibacterial effects of anthocyanins involve interactions with bacterial cell membranes, including hydrogen-binding membrane proteins and hydrophobic interactions [15, 28, 29]. Anthocyanins can affect membrane structure and the stability of proteins requiring ions, demonstrating electron donation or binding capabilities at the membrane interface, as supported by Lacombe *et al*. [27], Hosoda *et al*. [30], and Kwon *et al*. [31].

The higher SOD and TAC levels observed in the ANS group compared with the other groups suggest enhanced antioxidant capacity. This supports the hypothesis that anthocyanin-rich diets can mitigate oxidative stress in lactating goats. The antioxidant properties of anthocyanins in ANS likely contributed to improved health and potentially better production outcomes under heat stress conditions, which are common in tropical regions.

Onjai-uea *et al*. [15] reported that 100% purple NS treatment led to increased levels of 2,2-diphenyl-1-picrylhydrazyl, TAC, SOD, and glutathione S-transferase enzymes in both plasma and milk. Anthocyanins, which are flavonoids, can donate electrons in their natural form to ROS, effectively preventing the oxidation of biomolecules, such as polyunsaturated fatty acids, proteins, and DNA. In addition, anthocyanins exhibit metal-ion-chelating properties and act as free radical scavengers [10, 28].

## Conclusion

The inclusion of ANS in the diets of lactating crossbred Saanen goats offers multiple benefits, including comparable feed intake and milk production to traditional CS, while also enhancing antioxidant capacity. These findings suggest that ANS could be a valuable component of dairy goat diets, particularly in regions prone to heat-induced oxidative stress. Further research with larger sample sizes and varied environmental conditions is recommended to confirm these results and explore the long-term impacts of ANS on goat health and productivity.

## Authors’ Contributions

AC: Conducted feed analysis, investigated anthocyanins, assessed digestibility, and performed statistical analysis. JS, PS, PP, and WI: Coordinated the research and provided guidance on the study. JWL: Supervised the study and reviewed the initial draft of the manuscript. AT: Designed the experiments, conducted the research, including milk and blood collection and investigation, performed the statistical analysis, wrote the original draft, and edited the manuscript. All authors have read and approved the final manuscript.
